# Combined intranasal and intramuscular parainfluenza 5-, simian adenovirus ChAdOx1- and poxvirus MVA-vectored vaccines induce synergistically HIV-1-specific T cells in the mucosa

**DOI:** 10.3389/fimmu.2023.1186478

**Published:** 2023-07-17

**Authors:** Ashley C. Beavis, Edmund G. -T. Wee, Belkis M. Akis Yildirim, Nicola Borthwick, Biao He, Tomáš Hanke

**Affiliations:** ^1^ Department of Infectious Diseases, College of Veterinary Medicine, University of Georgia, Athens, GA, United States; ^2^ The Jenner Institute, Nuffield Department of Medicine, University of Oxford, Oxford, United Kingdom; ^3^ Joint Research Center for Human Retrovirus Infection, Kumamoto University, Kumamoto, Japan

**Keywords:** CD8+ T cells, HIV vaccine, ChAdOx1, MVA, PIV5

## Abstract

**Introduction:**

The primary goal of this work is to broaden and enhance the options for induction of protective CD8^+^ T cells against HIV-1 and respiratory pathogens.

**Methods:**

We explored the advantages of the parainfluenza virus 5 (PIV5) vector for delivery of pathogen-derived transgenes alone and in combination with the in-human potent regimen of simian adenovirus ChAdOx1 prime-poxvirus MVA boost delivering bi-valent mosaic of HIV-1 conserved regions designated HIVconsvX.

**Results:**

We showed in BALB/c mice that the PIV5 vector expressing the HIVconsvX immunogens could be readily incorporated with the other two vaccine modalities into a single regimen and that for specific vector combinations, mucosal CD8^+^ T-cell induction was enhanced synergistically by a combination of the intranasal and intramuscular routes of administration.

**Discussion:**

Encouraging safety and immunogenicity data from phase 1 human trials of ChAdOx1- and MVA-vectored vaccines for HIV-1, and PIV5-vectored vaccines for SARS-CoV-2 and respiratory syncytial virus pave the way for combining these vectors for HIV-1 and other indications in humans.

## Introduction

CD8^+^ T cells impose selective pressure on HIV-1 and their effector functions should be harnessed for vaccine protection (1–7). We aim to develop a vaccine strategy capable of inducing protective T-cell responses. This could reinforce antibody-mediated prevention and galvanize a cure for HIV-1. While our vaccine program is mainly informed by small rapid iterative studies in humans, animal models play an important part in the search for novel vaccine modalities, alternative delivery modes and their combinations. Our first clinically tested vaccine immunogen was termed HIVA and consisted of HIV clade A Gag p24 and p17 coupled to partially overlapping T-cell epitopes (8, 9). Following the field’s full appreciation of the enormous HIV-1 ability to change, the 1^st^-generation HIVconsv vaccines focused on the functionally conserved regions of the HIV-1 proteome with a high degree of similarity among all global variants (10, 11). This has now been replaced by 2^nd^-generation conserved mosaic immunogens HIVconsvX dealing more efficiently with the residual global HIV-1 diversity within the conserved vaccine regions (12). Vector delivery has progressed from weakly immunogenic DNA-poxvirus MVA of HIVA to the current simian adenovirus ChAdOx1-MVA regimen shown to induce robust T cells in humans (8, 10, 13, 14). Despite the ChAdOx1-MVA human potency, we continuously search for novel alternative vaccine platforms to extend options for HIVconsvX delivery, improve T-cell induction, boost protection in low responders, facilitate long-term maintenance of responses and gain access to anatomical niches of HIV-1 transmission and persistent replication. To date, peptide-pulsed dendritic cells (NCT03758625), integration-deficient dendritic cells-targeting lentivirus (15), BCG (16), self-amplifying mRNA of BioNTech (17) and mRNA formulated in lipid nanoparticles of Moderna (18) have been explored as vaccine modalities for the HIVconsvX immunogens, some of which have entered and others are in search of funds for clinical development.

As mucosal surfaces are the primary route of HIV-1 infection, it is likely that mucosa-associated immunity, both cellular and humoral, would provide additional protection against HIV-1. To induce a localized immune response, it is advantageous to administer vaccines at the site of pathogen infection (19). Vaginal, rectal, and intranasal routes of HIV vaccine delivery have been investigated for DNA, MVA, fowl poxvirus and bacterial vectors with varying levels of efficacy (20–22). A vector with natural mucosal tropism might outperform other non-mucosal vectors (19). Parainfluenza virus 5 (PIV5), formerly known as simian virus 5 (SV5), is a respiratory virus that utilizes sialic acid as its receptor. It has a non-segmented negative-sense RNA genome of 15,246 bases, which has 7 genes that encode 8 proteins (23). PIV5 can infect virtually any mammalian cell without causing cytopathic effect (24) and has not been linked to disease in any animals. The kennel cough vaccine contains live PIV5 and has been administered intranasally to dogs for over 40 years without causing any safety or environmental concerns. In additional to inducing systemic, antigen-specific humoral and cellular immunity, PIV5-vectored vaccines can induce mucosal IgA antibodies (25, 26). Furthermore, pre-existing anti-PIV5 antibodies do not interfere with the induction of immune responses to the transgene product (27). During a phase 1 clinical trial in the United States, PIV5 expressing SARS-CoV-2 spike protein was intranasally administered to humans and shown to be safe and immunogenic (NCT04954287; unpublished, B.H.), prompting approval of phase 2 clinical trial (NCT05736835). Additionally, an intranasal PIV5-vectored RSV vaccine showed excellent safety in the phase 1 clinical trial and was approved for a phase 1/2a clinical trial in infants (unpublished, B.H.; NCT05281263; NCT05655182). Collectively, these features support the use of engineered PIV5 as a vaccine vector with the potential to become one of the future broadly used ‘plug-in-and-go’ vaccine platforms.

In this work, we inserted the HIV-1 2^nd^-generation HIVconsvX conserved mosaic immunogens into PIV5 and characterized the transgene products expression and immunogenicity in a mouse model with emphasis on intranasal vaccine administration, which leads to efficient induction of HIV-1-specific T cells in the lungs and other mucosae. The ramification of these results for further HIVconsvX vaccine development is discussed.

## Materials and methods

### Cell lines

Human embryonic kidney 293T (HEK293T) cells were maintained in DMEM10 [Dulbecco’s modified Eagle medium supplemented with 10% fetal bovine serum (FBS), and 100 IU/ml penicillin, 100 µg/ml streptomycin (1% P/S) (Mediatech Inc., Manassas, VA, USA)]. Vero cells were maintained in DMEM5 supplemented with 5% FBS and 1% P/S. All cell lines were maintained at 37 °C with 5% CO_2_ and obtained from American Type Culture Collection (ATCC) (Manassas, VA, USA).

### Construction of the PIV5.HIVconsv2 and PIV5.HIVconsv5 vaccines

PIV5 strain canine parainfluenza (CPI) virus was used as the backbone for recombinant viruses P2 and P5. DNA fragments encoding for HIVconsv2 or HIVconsv5 were synthesized by Genscript (Piscataway, New Jersey, USA) and inserted as an additional open reading frame (ORF) between the PIV5 SH and HN ORFs as previously described (28). Briefly, to generate the recombinant P2 and P5 vaccines, plasmids encoding for the full-length genome cDNA of either P2 and P5, as well as helper plasmids pT7 polymerase, pPIV5-NP, pPIV5-P, and pPIV5-L, were co-transfected into HEK293T cells at 60-80% confluency in 6-cm^2^ plates. One day post-transfection, the HEK293T cells were trypsinized and combined with 10^6^ Vero cells in a 10-cm^2^ plate. After incubation at 37 °C, 5% CO_2_ for 7 days, the cell medium was harvested, and the viruses were plaque purified in Vero cells. For plaque purification, harvested cell medium was serially diluted and inoculated onto Vero cells, which were subsequently overlayed with 2% low melting point (LMP) agarose in DMEM5. After 7 to 14 days of incubation, individual plaques were picked and expanded in Vero cells. After 5 to 7 days, the cell medium was harvested, and cell debris was pelleted by centrifugation at 1000 rpm for 5 min. The supernatant was mixed with 0.1 volume of 10X sucrose-phosphate-glutamate (SPG) buffer and stored at -80 °C. Plaque assays were performed to titrate P2 and P5. Vero cells were infected with a serially diluted virus and overlayed with 2% LMP agarose in DMEM5. Following 10-14 days of incubation, the overlay was removed, the cells were fixed with 2% paraformaldehyde, and plaques were stained with 0.5% crystal violet in 25% methanol. The viral genome sequences of the full-length recombinant PIV5 vaccine viruses were confirmed to be correct with Sanger sequencing and expression of the transgene products by individual P2 and P5 viruses was confirmed.

### Immunofluorescence assay

To confirm HIVconsvX expression by the P2 and P5 vaccine viruses, Vero cells were infected at a multiplicity of infection (MOI) 3 and incubated at 37 °C, 5% CO_2_ for 2 days. The inoculum was removed, and the cells were washed with phosphate-buffered saline (PBS) and fixed with 60% methanol/40% acetone. The cells were then washed with PBS and incubated with 3% bovine serum albumin (BSA) in PBS for 30 min. Next, the cells were incubated for 1 hour with mouse anti-PIV5 V/P monoclonal antibody (mAb) and polyclonal rabbit anti-HIV-gag-p54-p24-p17 serum (Abcam catalogue #ab63917) diluted 1:200 in PBS plus 3% BSA. The cells were washed 3x with PBS and incubated for 30 min. with NucBlue Live Cell Stain (Invitrogen) and goat anti-mouse FITC mAb (Abcam) and goat anti-rabbit Cy3 (Fisher Scientific) serum diluted 1:200 in PBS plus 3% BSA. The cells were washed 3x with PBS and imaged with an Evos fluorescence microscope (Thermo Fisher Scientific, Waltham, MA, USA).

### Western blotting

To confirm HIVconsvX expression by the P2 and P5 vaccine viruses, Vero cells were infected at a multiplicity of infection (MOI) 3 and incubated at 37 °C, 5% CO_2_ for 2 days. Cell medium was collected and mixed with 2X Laemmle with 2-mercaptoethanol, and the cells were lysed in 1X Laemmle with 2-mercaptoethanol. The medium and cell lysate samples were incubated at 95 °C for 5 min. The samples were resolved with SDS-PAGE and transferred to Hamersham Hybond-LFP membranes (GE HealthCare Technologies Inc., Chicago, IL, USA). The membranes were incubated in PBS plus 5% non-fat milk for 1 hour. The membranes were washed with PBS plus 0.1% Tween-20 (PBST) and incubated for 1 hour with mouse anti-PIV5 V/P mAb or polyclonal rabbit anti-HIV-gag-p54-p24-p17 serum diluted 1:1000 in PBS plus 5% non-fat milk. Following washing with PBST, the membranes were incubated with anti-mouse or anti-rabbit Cy3 (Abcam) diluted 1:1000 in PBS plus 5% non-fat milk. The membranes were washed and imaged with a BioRad ChemiDoc MP Imaging System.

### Construction and preparation of the ChAdOx1- and MVA-vectored vaccines

Rescue strategies and preparation of the ChAdOx1.tHIVconsv1 (C1), ChADOx1.HIVconsv62 (C62), MVA.tHIVconsv3 (M3) and MVA.tHIVconsv4 (M4) vaccines were described previously (12).

### Animals and Immunizations

Groups of six-week-old female BALB/c mice were purchased from Envigo (UK) and vaccinated with the number of plaque-forming units (PFU) for parainfluenza viruses P2 and P5, and poxvirus M3 and M4 or virus particles (vp) of simian adenovirus C1 and C62 using either the intramuscular (IM) or intranasal (IN) route as indicated in each figure. Boost immunizations were delivered in 2-week intervals and the animals were killed 1 week after the last vaccine administration for the collection of the spleen, lungs, Peyer’s patches (PP) and female reproductive tract (FRT), the last two in selected experiments.

### Preparation of immune cells

Following vaccination, immune cells were isolated from the spleens, lungs, PP of the gut and FRT.

Immune cells from the spleen and PP were isolated by pressing harvested tissue through a 70-µm sterile nylon-mesh cell strainer (Thermo Fisher Scientific) using a 5-ml syringe rubber plunger. Following the removal of red blood cells with ACK lysis buffer (0.14 M NH_4_Cl, 10 mM KHCO_3_ and 100 mM Na_2_EDTA), cells were washed and resuspended in R10 (RPMI 1640 supplemented with 1% P/S, 10% FBS, and β-mercaptoethanol). Cells were counted using a CASY cell counter (Termo Fisher Scientific).

To obtain immune cells from the lungs and female genital tract, the dissected organs were cut into 1-mm^2^ segments and digested with 1.4 mg/ml Collagenase (Sigma Aldrich, Gillingham, UK) and 60 µg/ml DNase Type IV (Sigma Aldrich) in R0 supplemented with β-mercaptoethanol in 1.8-ml volume for 60 min. at 37 °C with shaking, after which 200 µl of FBS was added to quench the reaction. The cells were then processed in the same manner as those isolated from the spleen and Peyer’s patches.

### Peptides

H-2^d^ class I-restricted epitopes previously identified in the BALB/c mice and their variants (18, 29) were employed in immunological analyses. All peptides were at least 90% pure by mass spectrometry (Synpeptide, Shanghai, China) and were dissolved in DMSO (Sigma-Aldrich) to yield a stock of 10 mg/ml and stored at -80 °C until use. Aliquots were subsequently diluted to 1mg/ml with PBS and further diluted to working stock of 4-μg/ml concentration with R10, to give a final peptide concentration of 2 μg/ml when 50 μl of the peptide was assayed with 50 μl of cells in each well.

### IFN-γ ELISPOT assay

Interferon (IFN)-γ Enzyme-linked ImmunoSpot (ELISPOT) assay was performed using the Mouse IFN-γ ELISpot kit (Mabtech, Stockholm, Sweden) according to the manufacturer’s instructions as described previously (17). Briefly, immune cells were collected and tested separately from individual mice in triplicate wells. Peptides were used at 2 µg/ml each, and cells at 10^5^ cells/well were added to 96-well high-protein-binding Immobilon-P membrane plates (Millipore) that had been precoated with 5 µg/ml anti-IFN-γ mAb AN18 (Mabtech). For PP and FRT, all cells recovered were used and divided among the wells. The plates were incubated at 37 °C in 5% CO_2_ for 18 hours and washed with PBS before the addition of 1 µg/ml biotinylated anti-IFN-γ mAb (Mabtech) at room temperature for 2 hours. The plates were then washed with PBS, incubated with 1 µg/ml streptavidin-conjugated alkaline phosphatase (Mabtech) at room temperature for 1 hour, washed with PBS, and individual spot-producing units (SFU) were detected as dark spots after a 10-min reaction with 5-bromo-4-chloro-3-idolyl phosphate and nitro blue tetrazolium using an alkaline-phosphatase-conjugate substrate (Bio-Rad, Richmond, CA, USA). SFUs were counted using the AID ELISpot Reader System (Autoimmun Diagnostika, Strassberg, Germany). The frequencies of responding cells were expressed as SFU/10^6^ cells after subtracting the background frequencies from no-peptide assay wells.

### Statistical analysis

Statistical analyses were performed using Graph Pad Prism version 7.0. For ELISPOT data with 2 animals per group, average and individual animal values are shown. For more than 2 animals per group, non-parametric tests were used and median (range) is shown. For assessing the depth of variant peptide recognition, we used 2-way ANOVA with Tukey’s multiple comparisons tests. Two-tailed *P* values were used and *P* values of less than 0.05 were considered statistically significant.

## Results

### Vaccines

**Figure 1:**
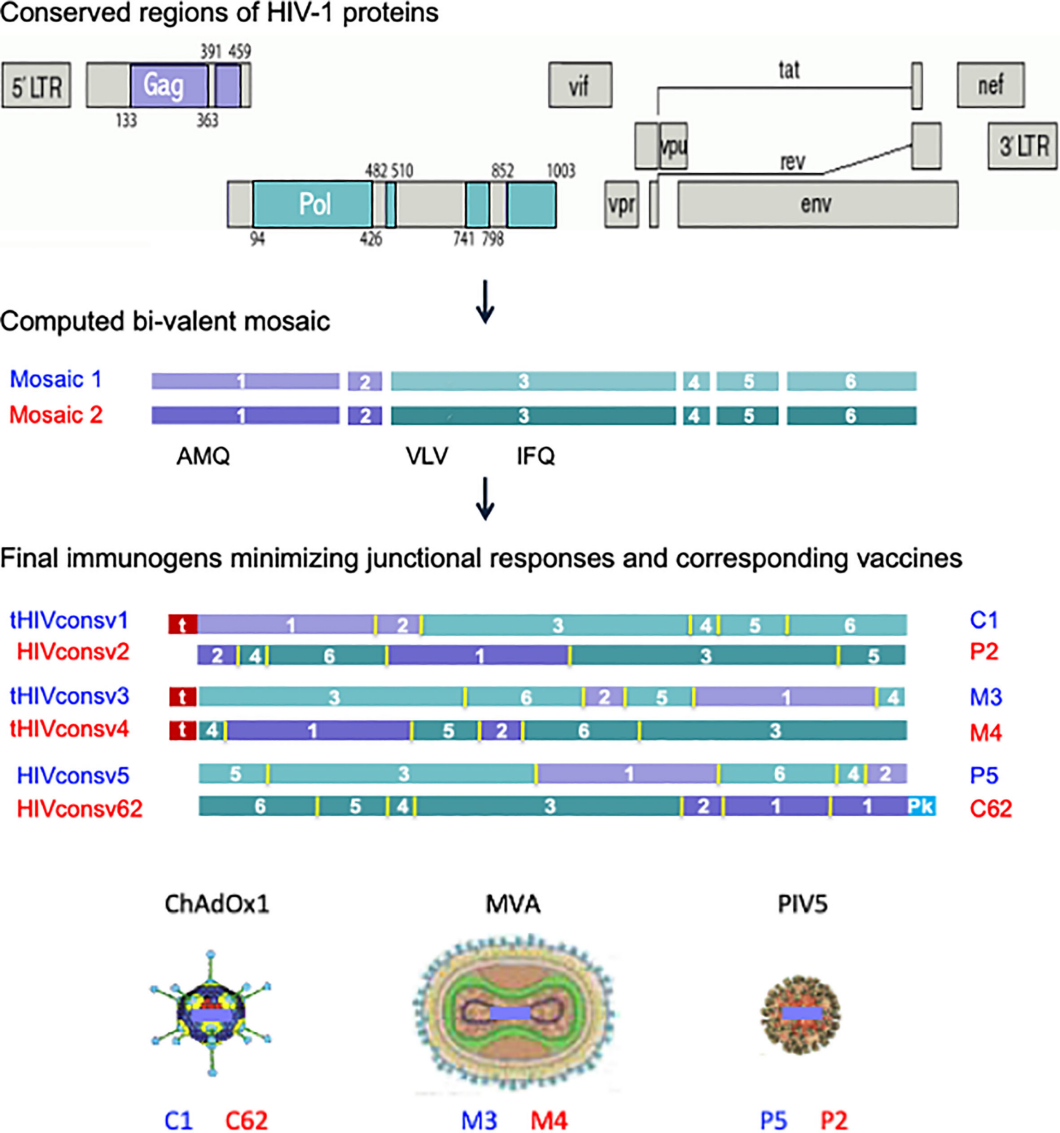
HIVconsvX immunogens and vaccines. Six conserved regions, two in the Gag and four in the Pol HIV proteins, of global HIV isolates of group M (top) were used to compute two mosaic versions of each for maximum vaccine match. The positions of three tested CD8+ T-cell epitopes (AMQMLKD/ETI, VLV/IGPTPVNI, and IFQS/CSMTKI) are indicated (middle). Regions were reshuffled to minimize responses to junctions and inserted into the ChAdOx1 vector derived from simian adenovirus, poxvirus MVA and parainfluenza virus 5, whereby N-terminal ‘t’ is for human tissue plasminogen activator leader sequence and C-terminal ‘Pk’ is for the SV5-P-k (aka SV5) monoclonal antibody tag, respectively (bottom). Note the three immunogens for each mosaic (HIVconsv1, HIVconsv3 and HIVconsv5 as mosaic 1 and HIVconsv2, HIVconsv4 and HIVconsv62 as mosaic 2) are identical for the amino acid and T-cell epitope content.

The 2^nd^-generation HIVconsvX bi-valent mosaic immunogens were derived from highly conserved regions of HIV-1 proteins Gag and Pol, while Env and other accessory proteins were purposely left out for lack of protective epitopes and/or sufficiently long stretch of conservation (12, 29). Mosaic 1 (odd vaccine numbers/blue) and mosaic 2 (even vaccine numbers/red) of HIVconsvX complement each other for the best coverage of potential 9-mer T-cell epitopes (PTE) of HIV-1 group M (29–32) and are intended to be used together for vaccination. Here, to evaluate the quality and location of elicited T-cells determined by the vaccine vector and route of administration rather than the T-cell depth of epitope variant recognition, mosaic 1 was employed alone for simplicity in all but the last figure. The vaccines utilized in this work were simian adenovirus-derived ChAdOx1.tHIVconsv1 (C1) and ChAdOx1.HIVconsv6.2 (C62), poxviruses MVA.tHIVconsv3 (M3) and MVA.tHIVconsv4 (M4), and parainfluenza virus 5 PIV5.HIVconsv5 (P5) and PIV5.HIVconsv2 (P2) (**Figure 1**).

**Figure 2:**
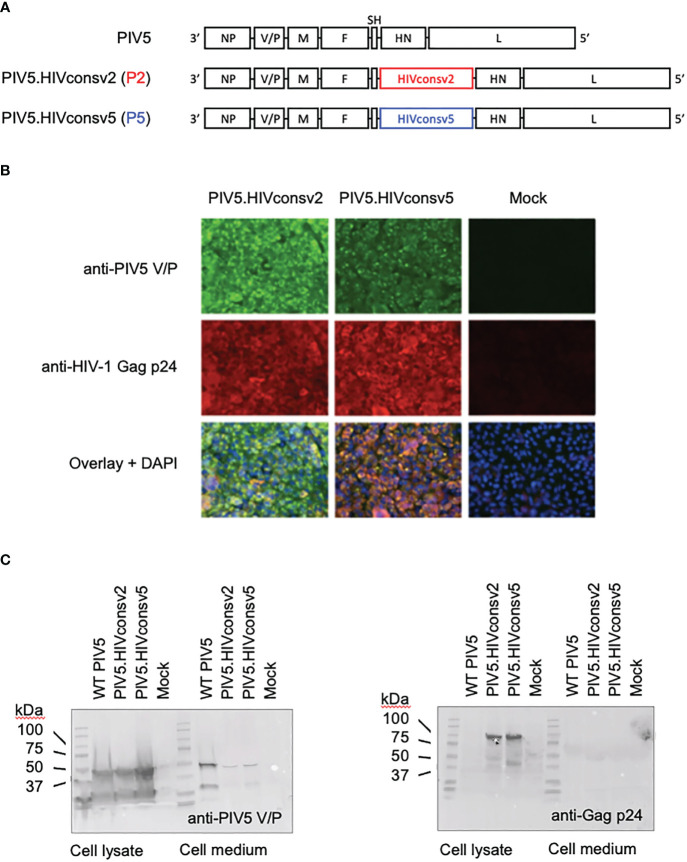
Construction of the PIV5.HIVconsv2 (P2) and PIV5.HIVconsv5 (P5). **(A)** The HIV-derived genes were inserted into the PIV5 genome between the PIV5 SH and HN genes. **(B)** Expression of the transgenes in infected Vero cells was readily detected by immunofluorescence using an anti-Gag p24 (region 1) mAb (red). Anti-PIV5 V/P protein mAb indicates the vaccine-infected cells (green). Cell nuclei were stained by DAPI. Western blot analysis of infected Vero cell lysates (left) and medium (right) was used to detect the PIV5 V/P protein **(C)** and confirmed expression of the correct full-length transgene HIVconsv2 and HIVconsv5 products of predicted 99.71 kDa and 98.81 kDa, respectively.

To construct parainfluenza vaccines P5 and P2, synthetic DNA fragments coding for the HIVconsv5 and HIVconsv2 immunogens were inserted between the SH and HN ORFs of the parainfluenza genome (**Figure 2A**). Abundant transgene expression was readily detected in infected Vero cells by immunofluorescence (**Figure 2B**) and Western blot analyses (**Figures 2C, D**), the latter also confirming the correct proteins predicted relative molecular masses.

### Two intranasal doses of PIV5.HIVconsv5 induce potent and broad T-cell responses in the lungs

**Figure 3:**
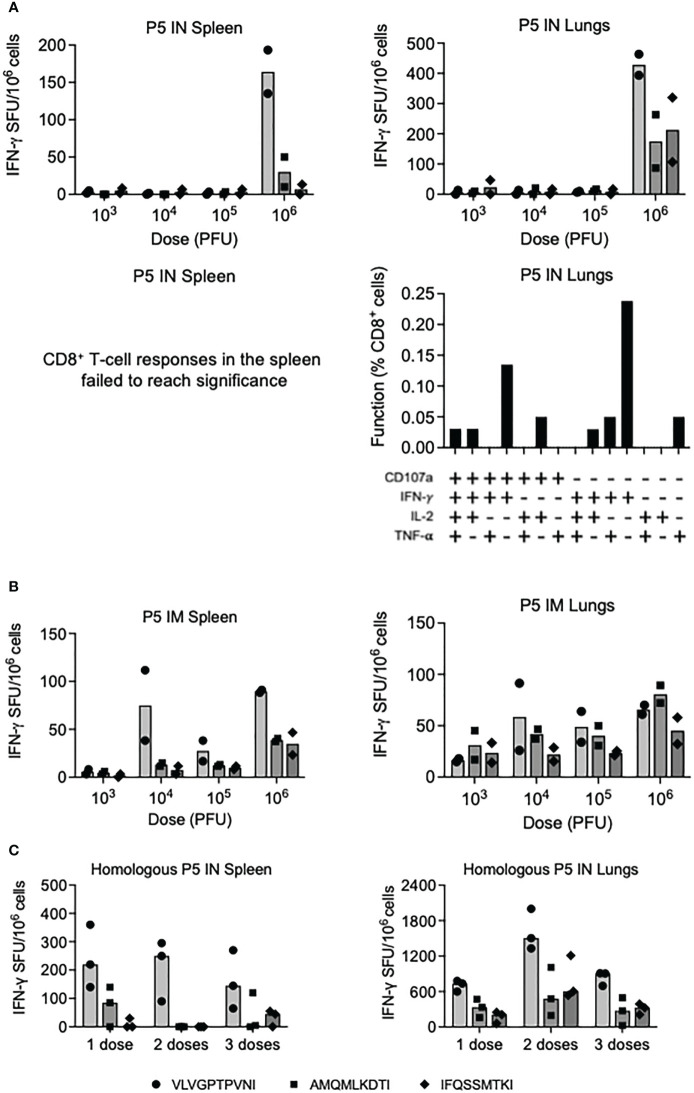
Induction of CD8^+^ T cells by homologous regimens of PIV5.HIVconsv5 (P5). Groups of BALB/c mice were immunized with increasing doses of the P5 vaccines either intranasally (IN) **(A)** or intramuscularly (IM) **(B)** and the frequencies of vaccine-elicited CD8^+^ T cells in the spleen (left) and lungs (right) were enumerated in an ELISPOT assay using the three strongest epitopes color coded as indicated on the bottom of the figure. Average (bars), as well as individual mouse frequencies, are shown (n = 2). For the IN delivery, the functionality of the VLV-specific T cells was determined (A bottom). The vaccine doses are shown in plaque-forming units (PFU). **(C)** One, two and three sequential IN administrations of the P5 vaccine were assessed in an ELISPOT assay and median (bars) as well individual mouse frequencies of elicited CD8^+^ T cells recognizing the three most immunodominant epitopes as indicated on the bottom of the figure were determined (n = 3).

Paramyxoviruses are respiratory viruses and therefore their natural intranasal administration (IN) was compared with intramuscular needle injection (IM) in a series of dose-response and repeated delivery experiments for induction of HIV-1-specific CD8^+^ T cells in the spleen and lungs. Using three well-defined H-2^d^-restricted CD8^+^ T-cell epitopes VLVGPTPVNI, AMQMLKDTI and IFQSSMTKI, listed in their immunodominance order, only the highest vaccine dose of 10^6^ plaque forming units (PFU) of P5 induced appreciable responses in an IFN-γ ELISPOT assay in both organs following IN delivery. Compared to the IFN-γ response in the spleens, the response in the lungs was approximately 3-fold higher and reached an average of 428 spot-forming units (SFU)/10^6^ cells against the immunodominant VLV peptide (**Figure 3A** top). In the lungs, the vaccine-elicited VLV-specific CD8^+^ T cells were polyfunctional and produced IFN-γ, tumor-necrosis factor (TNF)-α and interleukin (IL)-2. Furthermore, the cells degranulated as judged by surface expression of lysosomal-associated membrane protein (LAMP)-1, which is also known as CD107a (**Figure 3A** bottom). While IM delivery resulted in a better vaccine dose-increasing response, with up to an average of 81 SFU/10^6^ cells against AMQ, the overall response was lower in the lungs compared to IN vaccine delivery (**Figure 3B**). Finally, we tested multiple dosing of the same P5 vaccine IN and found that with 2-week intervals, two doses increased the single-dose frequencies of VLV-specific CD8^+^ T cells to median 250 and 1505 SFU/10^6^ cells in the spleen and lungs, respectively, while the third dose was less efficient (**Figure 3C**). Overall, the P5 vaccines were more immunogenic when delivered IN and induced potent, polyfunctional T cells in the lungs.

### Synergistic induction of T-cell responses in the lungs by a combination of PIV5.HIVconsv5 and ChAdOx1.tHIVconsv1

**Figure 4:**
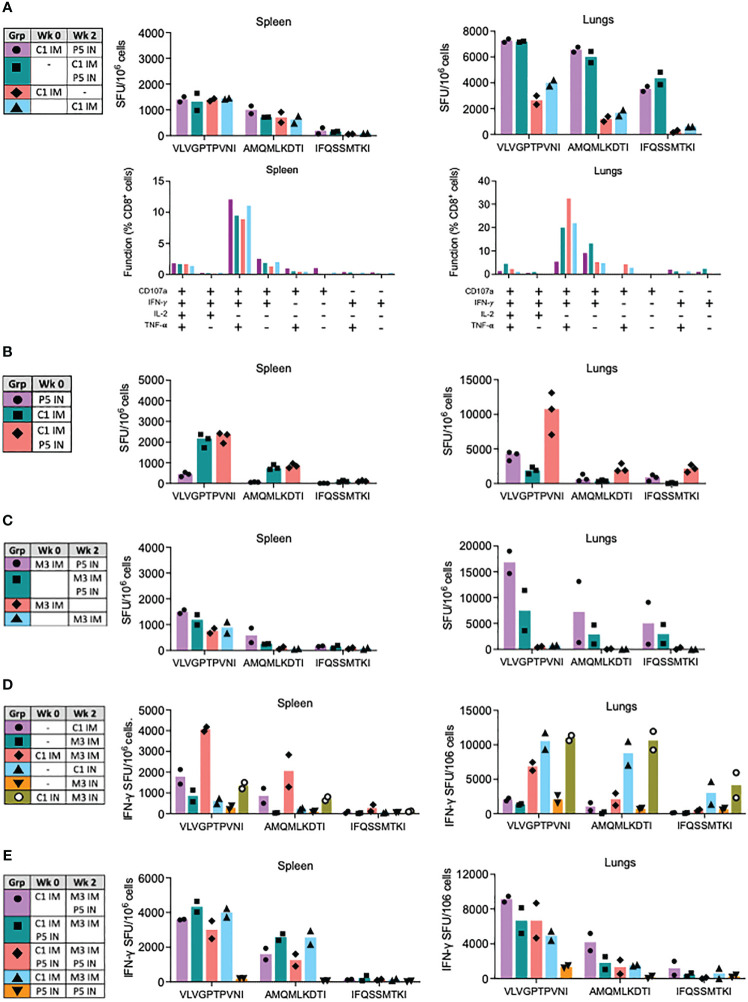
Synergy of ChAdOx1.HIVconsv1 (C1), MVA.tHIVconsv3 (M3) and PIV5.HIVconsv5 (P5) in heterologous regimens. BALB/c mice were immunized using regimens described and color coded in the tables (left). **(A)** Effect of the P5 IN boost was assessed in sequential and parallel administrations with C1 IM in spleens (left) and lungs (right) using the VLV, AMQ and IFQ peptides in an ELISPOT assay. The bars show average with individual mouse frequencies indicated (n = 2) (top). Functionality of the elicited responses was assessed in a polychromatic flow cytometry (bottom). **(B)** Parallel administrations of C1 IM and P5 IN were measured against each of the vaccines alone in the spleen and lungs. Heterologous regimens of ChAdOx1.HIVconsv1 (C1), MVA.tHIVconsv3 (M3) and PIV5.HIVconsv5 (P5) were tested next. **(C)** Effect of the P5 IN boost on sequential and parallel administrations with M3 IM. **(D)** Benefit of the sequential C1-M3 regimen delivered either IM or IN over each vaccine administered alone. **(E)** Optimizing triple delivery of C1, M3 and P5 in a single regimen. **(C)** Effect of the P5 IN boost on sequential and parallel administrations with M3 IM. The readout for all assays was an ELISPOT assay using the VLV, AMQ and IFQ peptides for restimulation. Median and individual animal values of T-cell frequencies are shown.

Next, we explored the vaccine modality of replication-deficient simian adenovirus Y25-derived vector ChAdOx1 for enhancement of the PIV5-induced CD8^+^ T-cell responses. First, we assessed the combination of ChAdOx1.tHIVconsv1 (C1) IM and P5 IN. While for splenocytes across all the tested regimens, there was a similar T-cell induction ranging from 1318 to 1448 SFU/10^6^ cells recognizing VLV, both sequential and parallel C1 IM and P5 IN delivery showed a synergistic effect on the frequencies of HIV-1-specific T cells in the lungs against all three VLV, AMQ and IFQ epitopes averaging up to 7265, 6568 and 4347 SFU/10^6^ cells, respectively. These cells were polyfunctional and, as expected for CD8^+^ T cells, the majority expressed IFN-γ, TNF-α and CD107a with minimal production of IL-2 (**Figure 4A**). Repeated parallel immunization compared the combined C1 IM and P5 IN vaccines to their individual administrations. Relative to C1 IM alone, similar levels of HIV-1-specific T cells in the spleens were induced by co-delivered C1P5 (median 2170 vs. 2365 SFU/10^6^ cells for VLV). However, synergism was confirmed for C1P5 vs P5 and C1 alone, stimulating respective medians of 10750 vs. 4300 and 1900 SFU/10^6^ lung cells for the immunodominant epitope. AMQ and IFQ displayed similar patterns, but with lower frequencies (**Figure 4B**).

### Fine-tuning of heterologous regimens of ChAdOx1.tHIVconsv1, MVA.tHIVconsv3 and PIV5.HIVconsv5 for lung CD8^+^ T-cell responses

In the next series of experiments, we explored the use of ChAdOx1.tHIVconsv1 and PIV5.HIVconsv5 with the poxvirus MVA.tHIVconsv3 (M3) vaccine. First, the benefit of P5 IN delivered with or after M3 IM was tested against M3 IM alone. The observations in the spleen were nonremarkable, and the splenocyte VLV T-cell frequencies were in the range of 1500 SFU/10^6^ cells, similar to C1P5. However, in the lungs, both the sequential and parallel regimens were again synergistic. Subsequent administration of M3 and P5 was over 2-fold more potent when compared to concurrent administration, and both administrations were superior to M3 alone, inducing an average of 16833, 7517 and ~715 VLV-specific SFU/10^6^ cells, respectively (**Figure 4C**). Being intrigued by the relatively unexplored IN delivery for C1 and M3, next, we compared C1M3 IN versus well-tested IM. The splenocyte results concurred with the well-described synergy between C1 and M3 IM, but less so for the IN route, eliciting 4075 and 1350 SFU/10^6^ splenocytes against VLV, respectively. In the lungs, C1M3 IM was synergistic and induced an average of 6850 SFU/10^6^ cells. In contrast to IM, for the IN route of delivery, C1 alone was almost as immunogenic as C1M3, while M3 IN was relatively much less immunogenic eliciting 10550, 10970 and 1500 SFU/10^6^ cells recognizing VLV, respectively (**Figure 4D**).

Finally, three vaccine platforms of simian adenovirus, poxvirus and PIV5 were combined into a single regimen to interrogate the possibility of enhancing the induction of HIVconsvX-specific CD8^+^ T cells even further. Thus, regimens C1-M3P5, C1P5-M3, C1P5-M3P5, C1-M3 and P5-P5 were compared, whereby C1 and M3 were always IM and P5 IN. In the spleen, the first four regimens induced VLV T-cell average in the range between 3003 and 4333 SFU/10^6^ splenocytes. In the lungs, these regimens’ averages arranged from the strongest to weakest aligned as C1-M3P5>C1P5-M3P5≅C1P5-M3>C1-M3, keeping in mind that only groups of 2 mice were tested due to the intense labor required for cell isolation from two organs in each animal (**Figure 4E**). Homologous P5-P5 IN was by far the weakest protocol. Again, the VLV immunogenicity patterns were closely matched by the subdominant AMQ and IFQ epitopes, but with lower frequencies.

### Induction of HIV-1-specific CD8^+^ T cells in diverse mucosa

**Figure 5:**
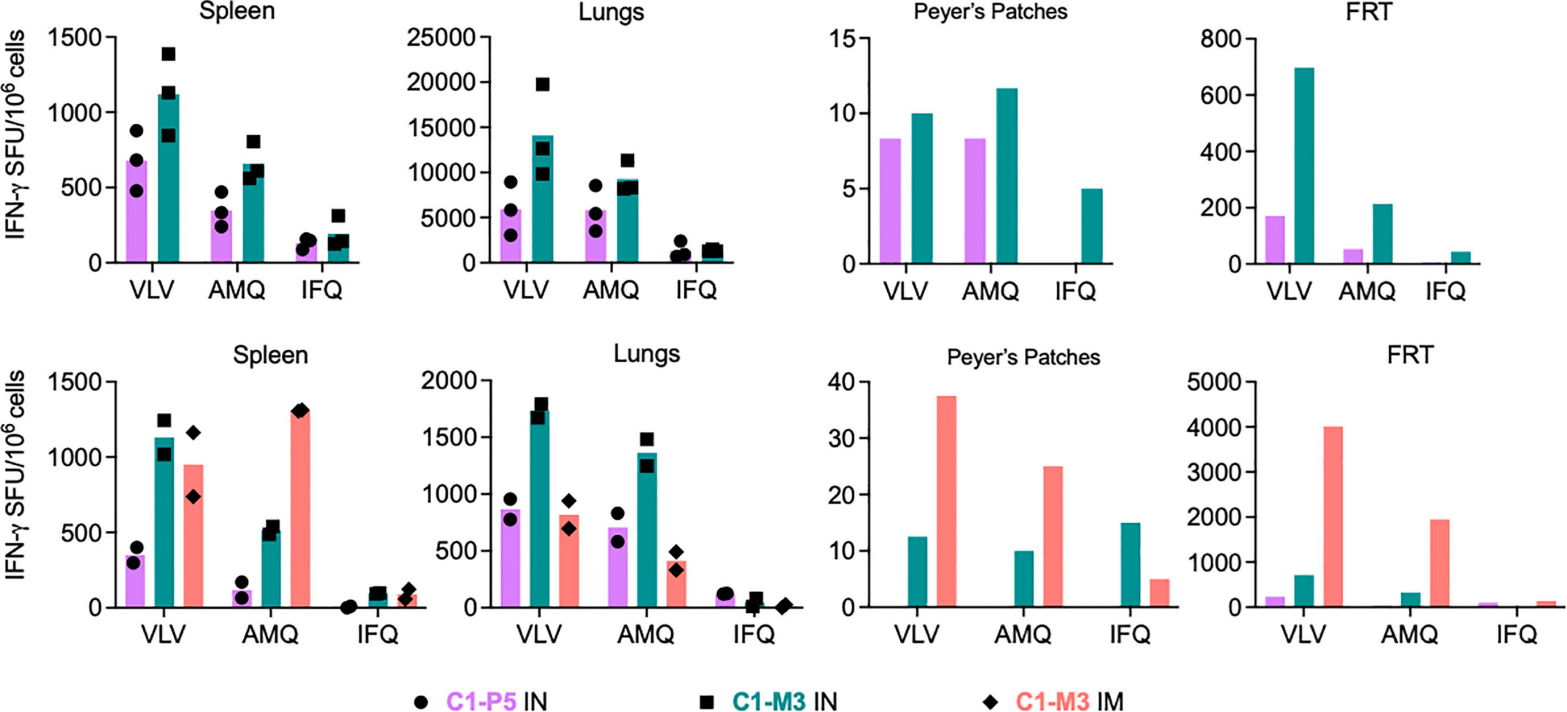
Induction of HIV-1-specific CD8^+^ T cells at mucosal sites. Two independent experiments are shown (top and bottom) of BALB/c mice immunizations using regimens of co-administered vaccines vie either the IN or IM routes as indicated below the graphs. Vaccine-elicited VLV-, AMQ- and IFQ-specific CD8^+^ T cells were enumerated in an ELISPOT assay in the spleen lungs, Peyer's patches (PP) and female reductive tract (FRT). For spleen and lungs, average (bars) and individual animal data (top n = 3 and bottom n=2) are shown. For PP and FRT, isolated immune cells were pooled prior to enumeration of HIV-1-specific CD8^+^ T cells.

Next, we investigated the induction of HIV-1-specific CD8^+^ T cells across diverse mucosal sites. Immune cells were isolated and tested from the lungs, Peyer’s patches (PP), the female reproductive tract (FRT), and the spleen as a reference. Isolation of CD8^+^ T cells from PP and FRT was labor-intensive, technically challenging and the cell yields were typically low. Therefore, for immunological analyses of PP and FRT, cells isolated from all animals in a group were pooled. For the pilot experiment, mice (n=3) were immunized using the C1-P5 IN and C1-M3 IN regimens, and the VLV-, AMQ- and IFQ-specific T-cell frequencies were determined in an IFN-γ ELISPOT assay. Both regimens induced frequencies concurrent with previous experiments in the spleen and lungs with median 679 and 1121 VLV-stimulated SFU/10^6^ splenocytes, and 5950 and 14083 SFU/10^6^ of lung cells for C1-P5 IN and C1-M3 IN, respectively. Marginal responses of 8 and 10 SFU/10^6^ cells above the background, respective to the regimens, were detected in PP, while 170 and 697 SFU/10^6^ cells responded in cells recovered from the FRT (**Figure 5** top). The same experiment (n=2) was repeated including a third group immunized C1-M1 IM. This time in all four organs, C1-M3 IN was better than C1-P5 IN with similar relative patterns of cell frequencies recognizing the VLV, AMQ and IFQ peptides. C1-M3 IM induced 950, 818, 38 and 4013 VLV-specific SFU/10^6^ cells in the spleen, lungs, PP and FRT, respectively. Compared to C1-P5 IN and C1-M3 IN, C1-M3 IM induced the most potent responses in PP and FRT (**Figure 5** bottom). Thus, C1C62-M3M4 IM tested currently in clinical studies remains a potent regimen, and its capacity to induce HIVconsvX-specific CD8^+^ T-cell effectors at the mucosal sites in humans should be assessed.

### Bi-valent PIV5.HIVconsv5+PIV5.HIVconsv2 (P5P2) IN vaccination induces ‘deep’ variant recognition

**Figure 6:**
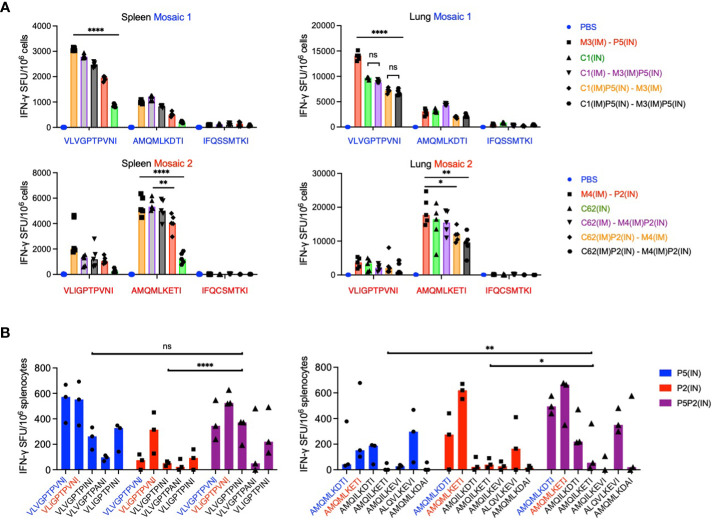
Benefit of the bi-valent mosaic design for epitope variant recognition. **(A)** Individual mosaic immunogenicities. In two separate experiments, groups of 5 BALB/c mice were immunized using the best three regimes for spleen and lungs with either mosaic 1 (top) or mosaic 2 (bottom) and the immune splenocytes were tested against mosaic-derived peptide variants. Median IFN-γ ELISPOT frequencies are depicted as bars with individual animal values shown. Dunn’s multiple comparisons tests were used to determine the significance of group differences for the most immunodominant epitope, which differed between the two mosaics. For Prism 9, P values are set at * - <0.05; ** - <0.01; and **** - <0.0001. **(B)** BALB/c mice were vaccinated with either mosaic 1 (P5), mosaic 2 (P2) or both mosaics together (P5P2) as two half doses, and the recognition of variant epitopes present in the vaccines as well as additional variants with ≥1% frequencies of HIV-1 isolates in the LANL-HSD was assessed for the VLV and AMQ epitopes in an ELISPOT assay. Results are shown as a median with individual values plotted (n = 3). 2-way ANOVA with Tukey’s multiple comparisons tests was used for the statistical analysis, whereby P values for AMQ are: ** - 0.0034 and * - 0.0232; and for VLV were ns – not significant 0.9932; and **** - <0.0001.

Next, we compared the immunogenicity of mosaic 2 (C62, M4 and P2) with mosaic 1 (C1, M3 and P5) using the strongest spleen and lung regimens, and groups of five mice to increase the power of our conclusions. These experiments broadly confirmed our previous regimen ranking. Direct comparison of the two mosaic performances is complicated by two individual experiments and the relative inter- and intra-immunogen dominance hierarchy of epitope variants present in the two immunogens (**Figure 6A**).

Finally, we evaluated the importance of the bi-valent mosaic design of HIVconsvX for induction of CD8^+^ T cells recognizing epitope variants beyond those present in the two vaccines, which we and others previously advocated (29, 30). Thus, immunization with mosaic 2 in P2 alone induced relatively poor responses to variant VLV peptides, which were substantially improved by mosaic 1 codelivery as P5P2. Furthermore, the recognition of variant AMQ peptides elicited by mosaic 1 of P5 benefited for at least five AMQ variants from the P5P2 bi-valent vaccination (**Figure 6B**). When the combined responses to both VLV and AMQ epitope variants were compared using 2-way ANOVA, the benefit of bi-valent vaccines was reached using Tukey’s multiple comparisons tests P values of 0.0138 for P5 vs. P5P2 and <0.0001 for P2 vs. P5P2. Thus, the BALB/c model affirms the induction of ‘deeper’ CD8^+^ T-cells by bi-valent vaccine administration, which may have important ramifications for the deployment of the HIVconsvX vaccines in an HLA outbred population exposed to diverse HIV-1, and the human PIV5 vectors can offer induction of such CD8^+^ T cells with improved depth of variant recognition.

## Discussion

In humans, the currently tested ‘core’ vector regimen aiming at induction of protective effector T cells against HIV-1/AIDS is ChAdOx1 prime and MVA boost delivering bi-valent conserved mosaic immunogens HIVconsvX (C1C62-M3M4) by the intramuscular route (10, 12). Promising data are supporting the benefits of narrowing the vaccine-elicited killer T cells towards specific sub-protein regions on HIV-1, such are the functionally conserved regions of HIVconsvX, as opposed to whole viral proteins (3, 7, 13, 33–35). However, functional features of vaccine-elicited CD8^+^ T cells that promote the prevention of HIV-1 acquisition and/or HIV-1 control after stopping antiretroviral therapy remain elusive (13, 34, 36–39). Hence, we continue to search for ways to improve CD8^+^ T-cell induction and ensure reaching, as yet undefined, protective numbers of T cells, which are polyfunctional, and display longevity, proliferative capacity, homing and any other parameters prerequisite for anti-HIV-1 immunity (40–46). Previous studies investigating the use of heterologous vectors showed that PIV5-vectored gp140 and SIV-Gag vaccines induced potent immune responses in rhesus macaques when boosted with a subsequent virus-like particle vaccine (26). In the present work, we constructed novel parainfluenza virus 5 (PIV5)-vectored vaccines and showed that these can be efficiently incorporated into immunization protocols with ChAdOx1 and MVA vaccines. Importantly, mixed routes of immunization and heterologous regimens, sequential and parallel, significantly potentiated vaccine induction of CD8^+^ T cells. We also demonstrated that both IN and IM routes of delivery could induce CD8^+^ T cells at lymphoid [spleen and Peyer’s patches (PP)] and mucosal [lungs and female reproductive tract (FRT)] sites.

Vaccine regimens are best compared in the same experiment with assays carried out on individual groups next to each other on the same day. This is challenging practically, especially when dealing with three vaccine modalities (PIV5, ChAdOx1 and MVA), two routes of delivery (IM and IN) and two, but sometimes four, anatomical sites in each animal (spleen, lungs, PP and FRT) to be analyzed. Thus, to visualize the relative efficiency of the vast number of regimens tested in this work, we ranked all the results in a single table based on descending average IFN-γ ELISPOT cell frequencies (**Table 1**). In the spleen, the most efficient vaccinations involved the core regimen C1(IM)-M3(IM) followed by all three combined modalities C1(IM)P5(IN)-M3(IM) and C1(IM)-M3(IM)P5(IN). In the lungs, rather unexpectedly, the leading vaccination was M3(IM)-P5(IN), followed by C1(IN)-M3(IN), single dose C1(IN), and C1(IM)-M3(IM)P5(IN) and C1(IM)P5(IN)-M3(IM)P5(IN). While we are not making any definite recommendations as to which of the tested regimens was the best, the underlying principles of combining routes and vaccine modalities to enhance potent induction of CD8^+^ T-cell responses systemically and at mucosal sites is worth exploring in humans. We also determined that overall, within the current set of experiments and several vaccine doses, mixed IM and IN route strategy induced the highest average frequencies of HIVconsvX-specific IFN-γ-producing T cells in the lungs (**Table 2**). It was noted that at two vaccine doses, the IM route alone induced higher frequencies of T cells in the lungs than the IN route alone and mixed three doses were the best suggesting that induction of systemic and mucosal T cells was not mutually exclusive nor heavily biased to one or the other. One limitation of our study design is that majority of the readout is dependent on IFN-γ production, which may especially in various organs, using IN and IM routes, and three vaccine modalities underestimate the true frequencies of vaccine-elicited HIVconsvX-specific CD8^+^ T cells. The frequencies of effector T cells in the mouse spleen or mucosa required to control or lessen the morbidity of a virus challenge likely differ for different viruses and T-cell specificities. Nevertheless, we find T-cell frequencies reaching 10-15 thousand cells per million quite impressive and comfortably superior to some previously reported frequencies protecting against experimental challenges (47–51).

**Table 1:** Regimens listed in order of the frequencies of vaccine-elicited CD8 T cells recognizing immunodominant epitope VLV.

Spleen				Lungs			
Prime	Boost		SFU/10^6^ cells	Prime	Boost		SFU/10^6^ cells
C1(IM)	M3(IM)		3855 (3/6)	M3(IM)	P5(IN)		14095 (2/7)
C1(IM)+P5(IN)	M3(IM)		3245 (2/7)	C1(IN)	M3(IN)		10600 (2/5)
C1(IM)	M3(IM)+P5(IN)		2821 (2/7)	C1(IN)			9746 (2/7)
C1(IM)+P5(IN)	M3(IM)+P5(IN)		2493 (2/7)	C1(IM)	M3(IM)+P5(IN)		8825 (2/7)
C1(IM)+P5(IN)			1943 (2/5)	C1(IM)+P5(IN)	M3(IM)+P5(IN)		8730 (2/7)
M3(IM)	P5(IN)		1787 (2/7)	M3(IM)+P5(IN)			7517 (1/2)
C1(IM)			1467 (4/8)	C1(IM)+P5(IN)	M3(IM)		7397 (2/7)
C1(IM)	P5(IN)		1414 (1/2)	C1(IM)	P5(IN)		7265 (1/2)
C1(IN)	M3(IN)		1200 (2/5)	C1(IM)+P5(IN)			7227 (2/5)
M3(IM)+P5(IN)			1189 (1/2)	C1(IM)	M3(IM)		4200 (3/7)
M3(IM)			765 (3/6)	C1(IN)	P5(IN)		3050 (2/4)
C1(IN)			811 (2/7)	C1(IM)			2708 (4/8)
C1(IN)	P5(IN)		478 (2/4)	M3(IN)			2050 (1/2)
P5(IN)			275 (2/5)	P5(IN)	P5(IN)		1468 (2/5)
M3(IN)			275 (1/2)	P5(IN)	P5(IN)	P5(IN)	905 (1/3)
P5(IN)	P5(IN)		187 (2/5)	M3(IM)			767 (3/6)
P5(IN)	P5(IN)	P5(IN)	145 (1/3)	P5(IN)			758 (3/8)
P5(IM)			90 (1/2)	P5(IM)			66 (1/2)

Data are shown as median IFN-γ-producing cell frequencies over all experiments in this manuscript (number of independent experiments/number of animals).

**Table 2:** Effect of the route of administration.

Spleen	1 dose	2 doses	3 doses	4 doses
IM	1347 (8/17)	3855 (3/6)	–	–
IN	345 (6/12)	683 (8/17)	145 (1/3)	–
MIXED IM/IN	–	1515 (5/11)	3817 (2/4)	3003 (1/2)
Lungs	1 doses	2 doses	3 doses	4 doses
IM	650 (8/17)	4934 (3/6)	–	–
IN	2050 (7/15)	2000 (8/17)	905 (1/3)	–
MIXED IM/IN	–	7397 (5/11)	8467 (2/4)	6669 (1/2)
Peyer’s patches	2 doses	FRT	2 doses	
IM	38 (1/2P)	IM	4013 (1/2P)	
IN	7 (2/4P)	IN	476 (2/4P)	

The numbers of doses indicate the total vaccine doses irrespective of the kind received by animals over the course of vaccination. Data are shown as median IFN-γ SFU/10^6^ cells (number of independent experiments/total number of mice) of T-cell responses to the VLV epitope; P – pooled cells from animals in the same group).

In the current study, we demonstrated the use of PIV5 as a vector platform for candidate HIV-1 vaccines inducing broad HIV-1-specific CD8^+^ T cells. Several previous publications supported the benefit of the bi- and indeed multi-valent HIVconsv/HIVconsvX designs on the ‘deeper’ recognition of variant HIV-1 epitopes of global HIV-1 isolates (18, 29, 30). In the present work, we reinforced these observations using the PIV5.HIVconsv5 (mosaic 1) and PIV5.HIVconsv2 (mosaic 2) vaccines together as P5P2(IN) as a stand-alone vaccine modality. Our results expand previously published increases in the levels of humoral and cellular responses against various pathogens by PIV5-based candidate vaccines in mice, hamsters, guinea pigs, ferrets, dogs, monkeys and most recently in humans (25, 27, 52–59). For the influenza virus, these immune responses protected mice and pigs against a virus challenge (60). Thus, through parental PIV5 safety, broad cell tropism, cross-species applicability, needle-free delivery, evasion of pre-existing anti-PIV5 immunity, and induction of CD8^+^ T cells, and mucosal and systemic antibodies, the PIV5 vector is a strong candidate for a versatile vaccine platform.

Evidence is emerging that tissue-resident memory CD8^+^ T (T_RM_) cells correlate with protection against infections, perhaps because they are typically in the mucosal epithelium and can activate earlier/faster than central memory T_CM_ cells. Thus, it was shown that intravaginal or intrarectal administration of candidate HIV-1 vaccines performed better compared to intranasal vaccination, with varying levels of immunity induced depending on the route of administration (20). While ChAdOx1-vectored Astra/Zeneca vaccine against SARS-CoV-2 applied using a nasal spray to humans was disappointing (61), this work demonstrated that ChAdOx1-vectored C1 vaccine induced impressive levels of T cells in mouse lungs. Across all the experimental designs and vaccine modalities, HIVconsvX vaccines induced 4.5-fold higher responses in the lungs than in the spleen (Student T Test P = 2.0x10^-16^) (**Table 1**). Although lungs are not directly relevant to HIV-1 transmission, intravaginal and/or intrarectal routes for mice vaccination are only being established in our laboratory, while IN is the natural route for parainfluenza virus ingress. It is an important and encouraging observation that the currently tested core regimen of C(IM)-M(IM) can induce mucosal-associated, HIV-1-specific cellular immunity. While most IN vaccines tested in human clinical trials are for respiratory viruses, there have been 4 trials for candidate HIV-1 vaccines administered IN: two peptide-based products Vacc-4x (62) and MYM-V101 (63), the latter delivered by IM/IN combination, human replication-competent adenovirus Ad4 Gag/Env boosted by protein Env (64) and canarypox ALVAC-HIV vCP205 (65). Our results suggest that the mixed IM/IN route of administration can have a synergistic effect on the induction of T cells in humans, too.

In conclusion, our study is rare in that it focuses on the induction of CD8^+^ T-cell responses rather than anti-HIV-1 antibodies. An effective vaccine against HIV-1 is long overdue and may eventually require concerted actions of antibodies and protective cells of both effector and resident memory. The long-term aim is to generate a large panel of vaccine modalities, which will be needed for the induction and maintenance of immunity against well-known and yet unidentified pathogens as well as for meeting demand for the global vaccine supply. The attractive properties of the parainfluenza virus 5 vector were supported and expanded by our current results, which put the PIV5 vector at the forefront with other promising delivery systems. All three vaccine vector modalities of ChAdOx1, MVA and PIV5 have shown promising safety and immunogenicity profiles in phase 1/2 vaccine trials in humans (T.H. and B.H., unpublished) warranting further studies examining their joint usage.

## Data availability statement

The original contributions presented in the study are included in the article/supplementary material. Further inquiries can be directed to the corresponding author.

## Ethics statement

All animal procedures and care were approved by the local Clinical Medicine Ethical Review Committee, University of Oxford and conformed strictly to the UK Home Office Guidelines under the Animals (Scientific Procedures) Act 1986. Experiments were conducted under project license PP1892852 held by TH.

## Author contributions

Conceptualization: TH and BH. Methodology: TH, EW, NB, AB, BA, and BH. Validation: EW. Investigation: AB, NB, and EW. Data curation: TH, EW, and NB. Writing—original draft preparation: TH. Writing—review and editing: all authors. Supervision: TH and BH. Project administration: TH. Funding acquisition: TH and BH. All authors have read and agreed to the revised version of the manuscript. All authors contributed to the article.
